# Modulation of growth and angiogenic potential of oral squamous carcinoma cells in vitro using salvianolic acid B

**DOI:** 10.1186/1472-6882-11-54

**Published:** 2011-07-05

**Authors:** Ya Yang, Ping J Ge, Long Jiang, Feng L Li, Qin Y Zhu

**Affiliations:** 1 Department of General Dentistry, Ninth People's Hospital, School of Stomatology, Shanghai Jiao Tong University School of Medicine, Shanghai Key Laboratory of Stomatology, 639 Zhi Zao Ju Road, Shanghai, 200011, China; 2 Department of Endodontics, Tongji Hospital of Stomatology, Tongji University, 399 Middle Yanchang Road, Shanghai, 200072, China

## Abstract

**Background:**

Our previous studies showed that Salvianolic acid B (Sal B) inhibited 7,12-dimethylbenz[a]anthracene (DMBA)-induced oral carcinogenesis in hamsters and such anti-cancer effects might be related to the inhibition of angiogenesis. This study was aimed to further investigate the anti-proliferative effect of Sal B on the most common type of oral cancer, oral squamous cell carcinoma (OSCC) and the possible mechanisms of action with respect to angiogenesis inhibition.

**Methods:**

Two well-characterized oral squamous cell carcinoma cell lines, CAL27 and SCC4, and premalignant leukoplakia cells were treated with different concentrations of Sal B. Cytotoxicity was assessed by MTT assay. cDNA microarray was utilized to evaluate the expression of 96 genes known to be involved in modulating the biological processes of angiogenesis. Real-time reverse transcription-polymerase chain reaction analysis was conducted to confirm the cDNA microarray data.

**Results:**

Sal B induced growth inhibition in OSCC cell lines but had limited effects on premalignant cells. A total of 17 genes showed a greater than 3-fold change when comparing Sal B treated OSCC cells to the control. Among these genes, HIF-1α, TNFα and MMP9 are specifically inhibited, expression of THBS2 was up-regulated.

**Conclusions:**

Sal B has inhibitory effect on OSCC cell growth. The antitumor effect can be attributed to anti-angiogenic potential induced by a decreased expression of some key regulator genes of angiogenesis. Sal B may be a promising modality for treating oral squamous cell carcinoma.

## Background

Carcinomas of the oral cavity, especially oral squamous cell carcinoma (OSCC), have become an important healthcare problem worldwide. Survival rate of oral carcinoma is lower than most other carcinomas, and has not been improved substantially in past years. The conventional treatment is a combination of surgery, radiation therapy and chemotherapy. Among these strategies, chemotherapy is beneficial for local control and survival improvement. Nevertheless, treatment with current chemotherapeutic drugs does not always substantially induce a positive response. Therefore, finding an effective therapeutic and preventive strategy for patients of such malignancy is of utmost importance. Screening Chinese medicine herbs or their extracts is believed a promising strategy to find effective chemopreventive agents.

Angiogenesis, the growth of new capillaries from preexisting blood vessels, is essential for cancer to grow beyond minimal size and metastasize [1,2]. Anti-angiogenesis remains a prime therapeutic target and anti-angiogenic therapy may be less susceptible to develop treatment resistance. Thus screening natural health products that inhibit angiogenesis is a potential source for investigating new agents to treat oral cancer.

*Salvia miltiorrhiza *(Danshen), a popular Chinese herb, has been widely and successfully used for treating angina pectoris, myocardial infarction (MI) and stroke [3]. Salvianolic acid B (Sal B), one of the major water-soluble compounds of Danshen, is the most abundant and bioactive member of the salvianolic acids in Danshen (exhibiting antioxidant, hepatoprotective and many other actions) [4]. Studies have shown that Sal B possesses many biological activities of the Danshen herb. For example, it was reported to possess anti-inflammatory and anti-oxidative properties, modulation of apoptosis, inhibition of platelet aggregation, improved coronary microcirculation [5-8]. It was also reported that Sal B enhanced angiogenic processes on SVR cells through up-regulation of VEGF and VEGF receptors genes [9], enhanced angiogenesis *in vitro *and improved skin flap survival in Sprague-Dawley rats [10], and improved the integrity of microvessels after ischemia [11]. But our previous studies showed that Sal B significantly decreased the squamous cell carcinoma (SCC) incidence from 64.7 (11/17) to 16.7% (3/18), with a simultaneous decrease in the immunostaining of HIF-1α and VEGF protein [12]. These findings suggest that Sal B may serve as a preventive and/or therapeutic agent against oral cancer. And inhibition of angiogenesis may be one of the mechanisms of action. This encouraging *in vivo *result prompted us to further investigate how Sal B affects oral squamous carcinoma cells growth and prohibits new vessels formation.

## Methods

### Drugs and Reagents

Sal B was a generous gift from Prof. Wei-dong Zhang, Department of Medicinal Chemistry of Nature Product, School of Pharmacy, Second Military Medical University, Shanghai, China. The purity of Sal B was 98% (determined by high-performance liquid chromatography method with fluorescence). Its molecular weight is 718, and its molecular formula is C_36_H_30_O_16_. Sal B was dissolved in double distilled H_2_O at a concentration of 200 mM, filtered through a 0.22 μm filter, and stored at -70°C. The stock solution was freshly diluted to the desired concentrations with medium immediately before use. Dulbecco's modified Eagle's medium (DMEM) and fetal bovine serum (FBS) were obtained from GIBCO BRL (Grand Island, NY, USA). 3'-(4,5-dimethylthiazol-2-yl)-2, 5-diphenyl tetrazolium bromide (MTT) was purchased from BD Pharmingen (USA).

### Cell lines

Three cell lines were used in this study, including CAL27, SCC4 and Leuk1. CAL27 and SCC4 were provided by Laboratory of Oral Tumor and Oral Biology, Shanghai Key Laboratory of Stomatology, Shanghai, China. Leuk1 was a gift from Prof. Li Mao of University of Maryland Dental School, Baltimore, USA. Leuk1 and CAL27 cells were cultured in DMEM (Invitrogen) medium supplemented with 10% (v/v) fetal bovine serum, 100 units/ml penicillin and 100 units/ml streptomycin (GIBCO). SCC4 cells were maintained in DMEM/F12 supplemented with 10% FBS, 100 units/ml penicillin and 100 units/ml streptomycin. All cells were maintained in a humidified atmosphere of 5% CO2 at 37°C.

### Cell viability assay

The inhibitory effect of Sal B on the cell viability was measured by MTT colorimetric method. Cells were plated into a 96-well plate at a density of 1 × 10^3^/well in 96-well tissue culture plates. On day two, cells were treated with increasing doses of Sal B (50, 100, 200 ug/ml) for 24 h, 48 h and 72 h. After drug treatment, attached cells were incubated with MTT (0.5 mg/ml, 1 h) for 4 hours and subsequently solubilized in DMSO. The absorbance of each well was measured using an enzyme-linked immunosorbent assay reader at 490 nm. Experiments were performed at least three times.

### Nucleic acid isolation and cDNA probe preparation

Total RNA was isolated from cell culture flasks using RNAbee (Biogenesis), purified using chloroform, precipitated using cold isopropanol, washed using 75% ethanol. The resulting RNA concentration was measured spectrophotometrically and the quality of RNA was confirmed in agarose gels. cDNA for each sample was obtained by RT-PCR from 500 μg RNA in the presence of Biotin-16-dUTP (Roche Cat. No.1-093-070). Briefly, RNAs were denatured at 70°C for 10 min and cDNAs were synthesized at 42°C by oligo-dT priming in a final volume of 30 μl. The labelled cDNAs were purified by spin column chromatography. Then, the biotin-labeled cDNA was fragmented by incubation in fragmentation buffer at 94°C for 5 min and chilled on ice.

### cDNA microarray analysis

GEArray Q Series Angiogenesis Gene Array HS-009 (SuperArray Bioscience), containing 96 genes known to be involved in modulating the biological processes of angiogenesis, was prehybridized at 68°C for at least 90 min before probe addition in hybridization buffer. Then, the fragmented labeled cDNA was applied to the buffer. Hybridization was performed at 60°C overnight in a rolling bottle. The arrays were washed twice with 2 × SSC and 0.5% SDS at 60°C for 30 min; followed by two stringent washes with 0.5 × SSC, 0.5% SDS at the same temperature and for the same length of time. Finally, damp arrays were sealed in plastic wrap and exposed to imaging plates (BASMP 2040S; Fuji, Nakamura, Japan) for 24 hours, which were then scanned with a HP GeneArray Scanner (Hewlett-Packard, Palo Alto, CA).

### Microarray data analysis

*GEArray Analyzer *software was used for background subtraction and data normalization. Each GEArrayTM Q Series membranes were spotted with negative controls (pUC 18 DNA and blanks) and housekeeping genes, including β-actin, GAPDH, cyclophinin A and ribosomal protein L13a. All raw signal intensities should be corrected for background by subtracting the minimum value to avoid the appearance of negative numbers. All signal intensities should also be normalized to that of a housekeeping gene. These corrected, normalized signals can then be used to estimate the relative abundance of particular transcripts.

### Real-time reverse transcription-polymerase chain reaction (RT-PCR) analysis

The total RNA prepared for microarray analysis was also used for RT-PCR analysis of selected genes. Total RNA (2 μg) from each sample was subjected to reverse transcription using a Superscript first strand cDNA synthesis kit (Invitrogen) according to the manufacturer's protocol. Real-time PCR reactions were then carried out in a 25 μL reaction mixture (1 μL of cDNA, 12.5 μL of 2X SYBR Green PCR Master Mix, 1 μL of 20 μM specific gene primer pair, 2.5 μL of PCR buffer, 3 μL of MgCL_2 _solution, 3 μL of dNTP solution, 3 units of Taq and 2 3 μL of H_2_O) in an Rotor-Gene 3000 Realtime PCR machine (Corbett Research). The PCR program was initiated by 2 min at 50°C and 10 min at 95°C before 40 thermal cycles, each of 20 s at 94°C and 30 s at 72°C. Data were analyzed according to the comparative cycle threshold (Ct) method and were normalized by β-actin expression in each sample. Melting curves for each PCR reaction were generated to ensure the purity of the amplification product.

### Statistical analysis

The significance of results obtained from the control and treated groups was analyzed using the paired Student's t-test. Means and standard deviations were calculated. *P *< 0.05 was regarded as statistically significant.

## Results

### Effects of Sal B on cell viability

**Figure 1:**
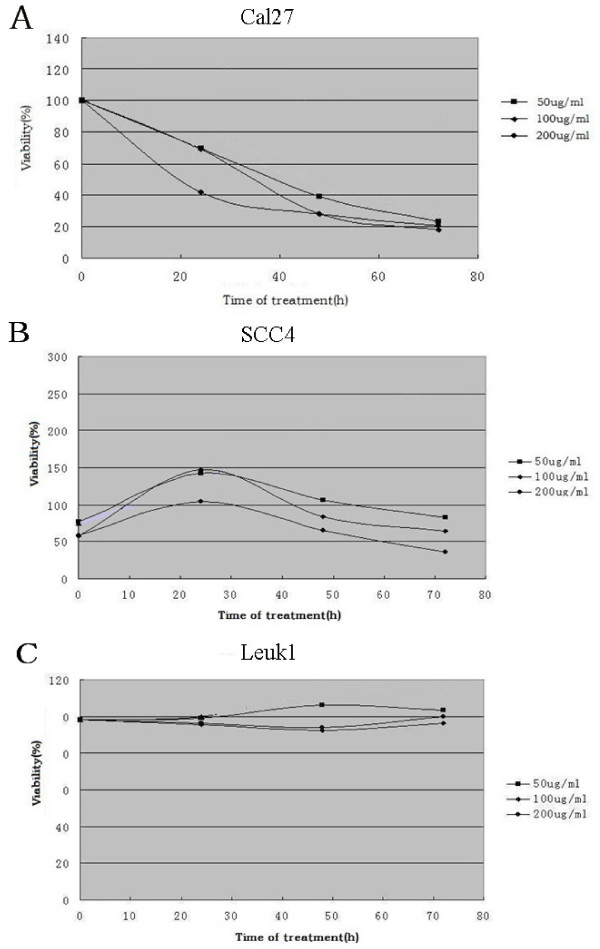
**Effects of Sal B on growth of human oral squamous cell carcinoma cells CAL27, SCC4 and oral precancerous cells Leuk1**. Cells (1 × 10^4^) were treated with increasing concentrations of Sal B (50, 100,200 μg/mL) for 72 h. Viable cells were measured by MTT assay and expressed as a percentage of control. All values are means of three independent experiments ± SD (*bars*). Time-dependent growth inhibition was seen in CAL27 cells. The SCC4 cells seemed to be stimulated after 24 h, but moderate inhibited at higher concentration after 48 h and inhibited after 72 h. No growth inhibition was also seen in Leuk1 cells. The significance of results obtained from the control and treated groups was analyzed using the paired Student's t-test. Means and standard deviations were calculated. *P *< 0.05 was regarded as statistically significant.

We evaluated the effects of Sal B on cell growth in the human oral squamous cell carcinoma cell lines CAL27, SCC4 and immortalized oral leukoplakia cell line Leuk1. Cells were incubated with increasing doses of Sal B (50, 100, 200 μM) or vehicle control for 24, 48 h and 72 h respectively; and cell viability was determined by a conventional tetrazolium-based (MTT) assay. As shown in Figure 1A and 1B, Time-dependent growth inhibition was seen in CAL27 cells. The SCC4 cells seemed to be stimulated after 24 hour, but moderate inhibited at higher concentration after 48 h and inhibited after 72 h. The IC_50 _values were 51 μg/ml and 87 μg/ml, respectively. In contrast, Sal B had a limited effect on the growth of Leuk1 (Figure 1C).

### Expression Profiling of Sal B treated cells

**Figure 2:**
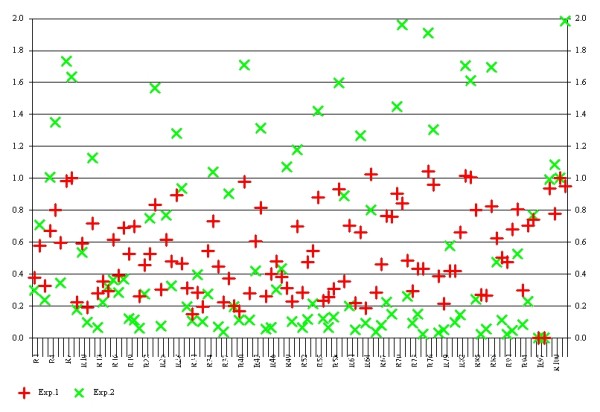
**The differential gene expression between the positive control and Sal B treated OSCC**. The crosses show the magnitude of differential expression between the positive control and Sal B treated OSCC. Red = the positive control; Green = Sal B treated OSCC.

We analyzed the gene expression profiles of Sal B treated CAL27 and SCC4. In comparison of Sal B treated cells with the control, we identified only two genes of which the scaled average difference values varied by ≥3-fold in both of the OSCC cell lines. One is down-regulated hypoxia inducing factor _1α _(HIF-1α), the other one is up-regulated Thrombospondin-2 (THBS2). Then we relaxed the stringency of our selection criteria to identify genes that exhibited a 3-fold expression difference in Sal B treated CAL27 or SCC4 cells relative to the control cells. The results demonstrated that 17 genes showing a greater than 3-fold change after Sal B treatment. Among these, 15 genes were down-regulated and 2 were up-regulated (Table 1, Table 2 and Figure 2). Genome profiles are available online through the NCBI Gene Expression Omnibus http://www.ncbi.nlm.nih.gov/geo/query/acc.cgi?acc=GSE29416SE29416.

**Table 1:** Angiogenesis-related Genes Significantly Affected by Sal B in CAL27 cells

	gene	code	Mean fold difference
**Down-regulated**	Tenascin _C_	NM_011607	10.41
	Osteopontin	NM_009263	8.50
	HIF-1_α_	NM_010431	8.19
	TGFb_1_	NM_011577	7.93
	Cox-2	NM_011198	7.61
	HGF	XM_131908	5.99
	Scya_2_	NM_011333	5.15
	IL-10	NM_010548	4.50
	TGFbR_2_	NM_009371	3.55
	Mmp_2_	NM_011697	3.48

***Up-regulated***	THBS2	NM_011581	3.42

**Table 2:** Angiogenesis-related Genes Significantly Affected by Sal B in SCC4 cells

	gene	code	Mean fold difference
**Down-regulated**	HIF-1_α_	NM_010431	5.75
	Mmp_9_		4.17
	TGF b_3_	NM_013599	4.01
	VEGF		3.42
	VEGF-C	NM_009368	3.21
	TNFa		3.05
		NM_009505	
		NM_009506	
		NM_013693	

***Up-regulated***	THBS2	NM_011581	3.23
	Timp1	NM_011593	3.15

### Target verification by RT-PCR

**Figure 3:**
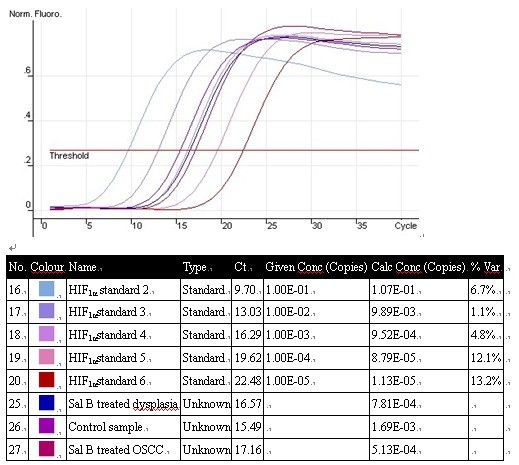
**Amplification plots of HIF-1_α _by quantitative real-time PCR**.

To verify the alterations of gene expression at the mRNA level, which appeared on the microarray, we chose four genes (HIF_1α_, TNF_α_, MMP_9, _THBS2) with varying expression profiles for real-time RT-PCR analysis. The results of real-time RT-PCR analysis for these selected genes were consistent with the microarray data. Gene expression alterations were similar by real-time RT-PCR analysis, although the fold changes in the expression level differed somewhat in the two analytical methods (Figure 3).

## Discussion

Clinical practice for years has proven that Salvia miltiorrhiza has anti-cancer potential and its application in the treatment of a variety of cancers has achieved surprising effects [13,14]. However, most of the studies about Sal B, one of the major biologically active components of Salvia miltiorrhiza, were focused on its effects on cardiovascular disorders. There are few data available about the effect of this component on cancer. It was for this reason that Sal B was evaluated in our study for its chemopreventive and chemotherapeutic potential against OSCC.

In the present study we investigated whether Sal B has any inhibitory properties on OSCC cells and potentially malignant Leuk1 cells, in order to provide more documentation on the possible application of Sal B on OSCCs and oral premalignant lesions. We have shown that Sal B potently inhibited cell growth and induced apoptosis in OSCC cell lines CAL27 and SCC4. However, Sal B was much less active in Leuk1. The mechanisms accounting for this selective growth inhibition need to be further investigated. It needs to be noted that Sal B undergoes degradation in normal saline solution which will affect its anti-cancer effect. But we found in this study that Sal B still significantly inhibited the growth of CAL27 and SCC4 cells after treatment over 24 hours. We suppose its anti-cancer effect will be more potent if Sal B could be used in a solid state.

Results of another study from this laboratory showed that the growth inhibitory effect of Sal B was related to the apoptosis-inducing effect of Sal B (determined by flow cytometry analysis, data not shown). The detailed mechanism(s) of the pro-apoptotic effect of Sal B on OSCC cells remains unclear, as we have not attempted to investigate this aspect of action in this study. But this finding, together with our previous *in vivo *results, suggests that Sal B may be a good candidate for therapy in patients with these malignancies.

Further microarray-based expression profiling and quantitative RT-PCR analyses were performed to confirm whether anti-angiogenesis is one of the possible mechanisms of Sal B-induced growth inhibition in CAL27 and SCC4 cells, the two OSCC cell lines sensitive to the growth inhibitory effects of Sal B. The results showed that HIF-1α is down-regulated by ≥3-fold in both of the Sal B treated CAL27 cells and SCC4 cells. This result was consistent with our previous immunostaining studies. In our previous study, we observed that the formation of microvessels, as well as the expression of pro-angiogenic factors HIF-1α and VEGF, was inhibited in dysplasia and SCC by Sal B [12]. HIF-1α is a transcription factor activated in response to cellular hypoxia. Being stabilized under decreased tissue oxygen concentration, it works as a cellular oxygen-sensing system, and trans-activates a large number of genes. Included among these are erythropoietin, glucose transporters, glycolytic pathway enzymes, and inducible nitric oxide synthase [15-17]. Discoveries have shown that hypoxia activates HIF-1α, which functions as master switches to induce expression of several angiogenic factors including VEGF, nitric oxide synthase (NOS), platelet-derived growth factor (PDGF) and Ang2. Alteration and over-expression of HIF-1α has been detected in a variety of solid tumors, including breast, lung, ovarian and oral cancer [18,19]. These observations, together with our results, strongly implied that inhibition of HIF-1α activation by Sal B, which resulted in lowered expression of downstream pro-angiogenic genes, may be a key mechanism of cell growth inhibition and anti-angiogenesis on oral cancers. Their relative protein expression levels and whether such an action can be demonstrated *in vivo *remains to be confirmed.

However, the anti-angiogenic effect of Sal B seemed contradictive to some previous studies which showed that Sal B might improve microcirculation by augmenting VEGF expression and promoting angiogenesis. [9-11,20]. Thrombospondins (TSPs) are known to inhibit neovascularization by induction of endothelial cell apoptosis through interaction with CD36 [21], inhibition of metalloproteinase activity [22], and inhibition of cell-cycle progression [23]. In addition to these well-known effects on endothelial cell proliferation and apoptosis, maintenance of vascular integrity is another notable function of TSPs [24-26]. In this study, thrombospondin-2 (THBS2) expression was up-regulated by ≥3-fold in both of the Sal B treated CAL27 cells and SCC4 cells. Another study in our lab showed that in the Sal B treated samples, the mural cell coverage index was significantly higher than that of the control. And the organization of mural cells in the two groups of samples was dramatically different (data not shown). This suggests that Sal B may prevent the formation of new vessels by promoting vascular maturation. It is possible that the pro-angiogenic effect of Sal B induces formation of mature vessels with efficient irrigation function. The new vessels are different from the angiogenesis in tumors which leads to the formation of a poorly organized vasculature characterized by tortuous and leaky vessels unable to support efficient blood flow (Further investigation will carried out to clarify this point). The potent effect of Sal B on blood circulation may reduce the hypoxia stress in the local tissues, thus inhibit the uncontrolled formation of leaky vasculature. Based upon this information, the current results may be in line with those of previous studies.

Several angiogenesis-associated genes, including Tenascin-C, Osteopontin, TGF-_β1_, Cox-2, HGF, MMP-_2 _and MMP-9 also displayed variable expression in this study. Expression of these genes was changed ≥3-fold in CAL27 cells or SCC4 cells. This result was consistent with earlier studies which reported that Sal B attenuates LPS-induced Cox-2, MMP-2 and MMP-9 expression in human aortic smooth muscle cells. [27,28] We found that these genes also play important roles in other ways that can affect cell signaling, the apoptotic pathway, cell metastasis, and other cellular behaviors. For example, expression of COX-2 was inhibited in Sal B treated OSCC cells. Over-expression of COX-2 is thought to contribute to carcinogenesis by stimulating cell proliferation [29], inhibiting apoptosis [30], and enhancing angiogenesis [31]. Such genes as Tenascin-C, Osteopontin and MMP, which were reported to contribute to tumor metastasis [32-35], were inhibited by Sal B in this study. Therefore, this suggested that Sal B may exert multiple effects on oral carcinogenesis. Further research is required to investigate whether other mechanisms, as anti-metastasis, anti-oxidant and anticoagulation effect, will contribute towards the chemopreventive effect of Sal B on OSCC cells.

## Conclusion

Our study suggests that Sal B has cytotoxic effect on OSCC cells. Its antitumor effect could be attributed to its anti-angiogeneic effect. Sal B may function by inhibiting expressions of such angiogenesis-associated genes as HIF-1α, THBS2, Tenascin-C, Osteopontin, TGFb1, Cox-2, HGF, and MMP_2_. Translational investigations to determine whether these angiogenesis-associated genes are regulated by Sal B in OSCC tumors *in vivo *and whether such regulations correlate with clinical response should further elucidate the mechanisms of action of this new agent in OSCC.

## Abbreviations

DMBA: 7,12-dimethylbenz[a]anthracene; OSCC: oral squamous cell carcinoma; Sal B: Salvianolic acid B; MVD: microvessel density; HIF-1α: Hypoxia inducible factor 1, alpha subunit; TNFα: tumor necrosis factor-α; VEGF: vascular endothelium growth factor; MMP9: Matrix metalloproteinase 9; COX-2: Cyclooxygenase 2; TSPs: Thrombospondins; THBS2: Thrombospondin 2

## Competing interests

The authors declare that they have no competing interests.

## Authors' contributions

YY and QZ were responsible for the study design, interpretation of the data and revision of the manuscript. YY and PG carried out the experimental work, LJ and FL did the statistical analysis. YY and PG prepared the manuscript, QZ made critical revisions. All authors read and approved of the final manuscript.

## Pre-publication history

The pre-publication history for this paper can be accessed here:

http://www.biomedcentral.com/1472-6882/11/54/prepub
